# Rare Finding of Inflammatory Fibroid Polyp of the Duodenum: A Complete Diagnostic and Pathological Workup

**DOI:** 10.7759/cureus.16745

**Published:** 2021-07-30

**Authors:** Aleksandar Zlatarov, Nadezhda Stefanova, Stefan Mihaylov, Doroteya Malinova

**Affiliations:** 1 Department of General and Operative Surgery, Medical University "Prof. Dr. Paraskev Stoyanov", Varna, BGR; 2 Department of General and Clinical Pathology, Forensic Medicine and Deontology, Medical University "Prof. Dr. Paraskev Stoyanov", Varna, BGR

**Keywords:** inflammatory fibroid polyp, duodenum, immunohistochemistry, duodenal polyp, differential diagnosis of duodenal tumor

## Abstract

Inflammatory fibroid polyps (IFP) are solitary benign tumors rarely found in the gastrointestinal (GI) tract. Additionally, duodenal polyps are diagnosed incidentally. We present a case of a 51-year-old female admitted to the department with an initial diagnosis of duodenal polyp on gastroscopy, CT, and positron-emission tomography (PET). COVID-19 pandemics was the reason for delayed treatment which allowed the lesion to progress and almost double its size in an eight-month period. We performed conventional duodenotomy and excision of the polyp. Diseases like gastrointestinal stromal tumor (GIST), inflammatory myofibroblastic tumor, and inflammatory polyp of Crohn’s disease must be considered in the differential diagnosis of IFP because they could be observed in the same location.

## Introduction

Inflammatory fibroid polyps (IFP) are solitary benign tumors rarely found in the gastrointestinal (GI) tract. They typically originate from the submucosal layer without encapsulation. The polyp structure consists mostly of loose connective tissue, inflammatory eosinophilic infiltrate, and blood vessels. The pathogenesis is unknown. It is believed that reactive response to unknown irritating stimuli is in the depth of this benign lesion. Rapid growth is often seen in this type of polyps. Immunohistochemical analysis suggests that this lesion is a true tumor and possibly with myofibroblastic differentiation. It is most common among adults in the 5th to 7th decade of life and does not have a preferred gender. Occurrence in children is rare. It is located mostly in stomach 70%; small bowel (ileum) 23%; colon 4%; gallbladder 1%; esophagus 1%; duodenum 1%; appendix <1%. Clinical presentation is according to the site of the GI tract that is involved. Usually, they are asymptomatic and develop for long periods. Symptoms vary from non-specific abdominal pain to upper GI blood loss, weight loss, dyspepsia, or symptoms suggesting obstruction.

## Case presentation

**Figure 1:**
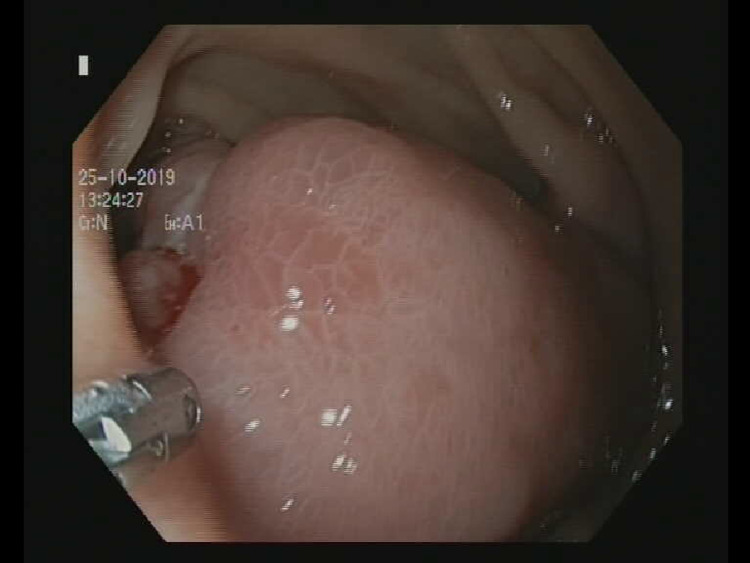
Endoscopic image of the duodenal polyp.

A 51-year-old female presented to the department with complaints of chronic fatigue, laboratory findings of microcytic hypochromic anemia, and hemoglobin level of 66 g/l. The patient neither presented with additional GI complaints nor had signs of GI hemorrhage. The esophago-gastro-duodenoscopy (EGD) revealed a pedunculated polyp in the descending part of the duodenum with a small area of superficial ulcerated mucosa (Figure 1). The endoscopic biopsy showed duodenal mucosal epithelium with minor signs of dysplasia, solitary mucous glands, eosinophilic inflammatory infiltrate, and granulation tissue proliferation. This result neither suggested epithelial origin, nor could rule out malignant origin and the patient was scheduled for endoscopic ultrasound and CT.

**Figure 2:**
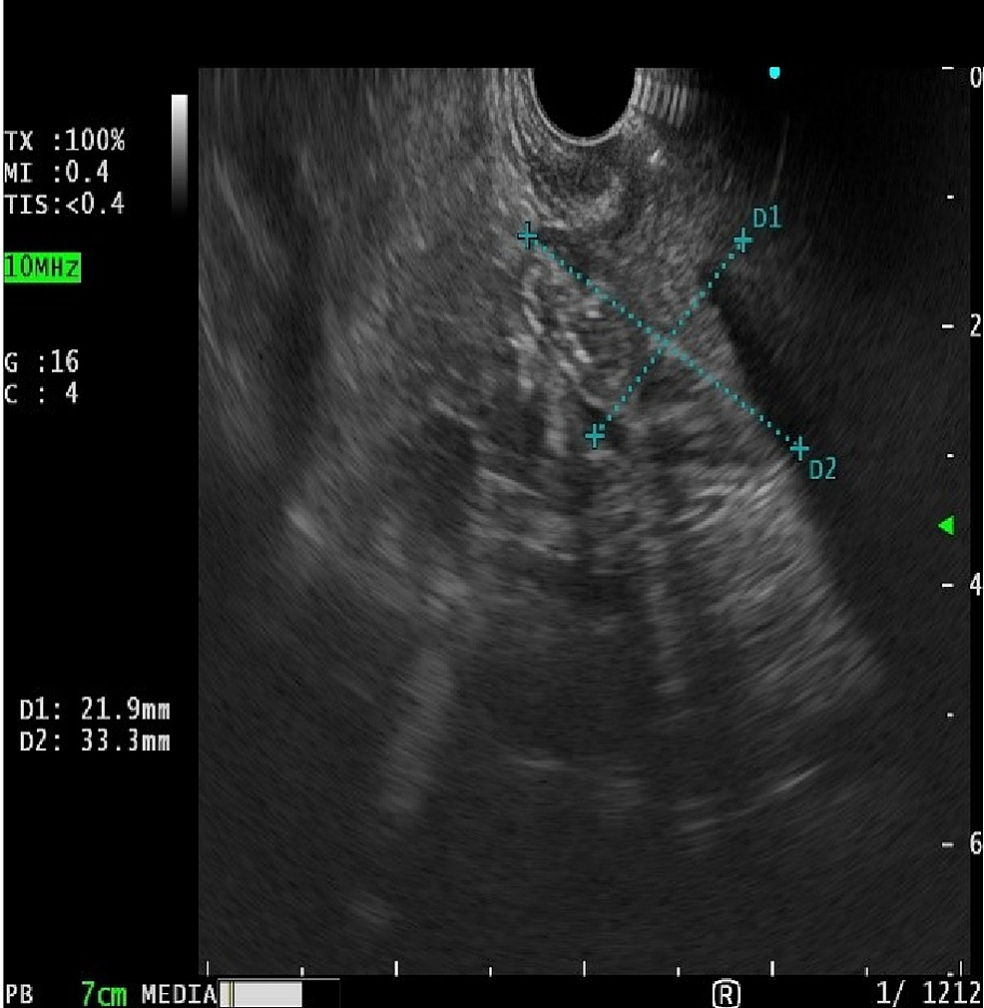
Endoscopic ultrasonography (EUS) revealed tumor mass originating from the second ultrasound layer with heterogenous isoechoic structure.

Endoscopic ultrasound revealed a lesion in the second sonographic layer of the duodenal wall without distinct margin and a heterogenous isoechoic echo pattern (Figure 2).

**Figure 3:**
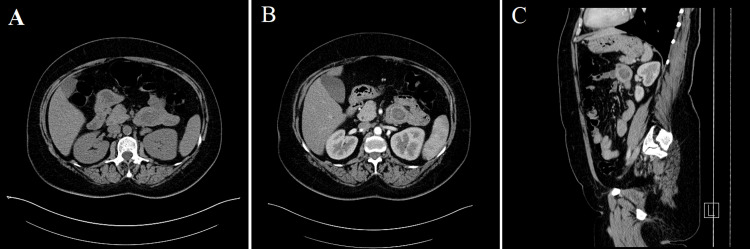
Computer tomography image of the duоdenal polyp localized in the horizontal and descending part of duodenum with peripheral enhancing of 18-20 HU and hypodense core. A - native image, transversal plane. B - arterial contrast phase, transversal plane. C - arterial contrast phase, saggital plane.

The CT confirmed pedunculated polyp sized 51 x 27 x 22 mm in the horizontal and descending part of duodenum with peripheral enhancing of 18-20 HU and hypodense core (Figure 3). No abdominal lymphadenopathy was found. 

The patient had significant comorbidities such as ischemic heart disease with stenting of two coronary branches, hypertension, and chronic obstructive pulmonary disease. Risk factors include smoking over 40 years, uncontrolled obesity with body mass index (BMI) of 31.0. The patient was scheduled for elective surgery, but due to the COVID-19 pandemics and the cancelation of elective surgical procedures, the treatment was postponed.

The patient failed to show up at appointments and sought medical attention eight months later with the progression of the initial symptoms and pronounced fatigability. Furthermore, the lack of physical activity led to an increase of the body weight and BMI to 33.7. The laboratory test confirmed again anemia and hemoglobin level 91 g/l. EGD and CT were repeated and demonstrated growth of the tumor which doubled its size to 85 x 30 x 28 mm. EUS showed intramural cystic transformation. Additional fluorodeoxyglucose (FDG)-positron emission tomography (PET) was performed, which did not establish increased metabolic activity of the lesion. Elective surgery was rescheduled.

The patient underwent laparotomy, duodenotomy, and excision of the polyp. The defect was closed with hand-sewn Heineke-Mikulicz duodenoplasty. No postoperative adverse effects and complications were registered.

**Figure 4:**
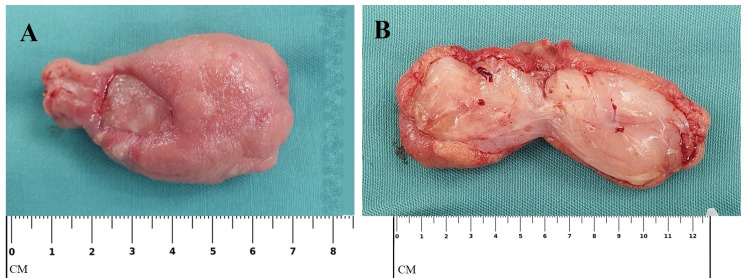
Gross pathological image of the polyp. Note the erosion site near the pedicle of the polyp. A - polyp as a whole. B - cross section of the polyp.

The gross pathological description was of a lesion of the duodenum with dimensions 55 x 40 x 30 mm with irregular, lobulated surface and swollen, thickened wall, in the submucosa there was a rounded mass with light, tan-white color (Figure 4).

**Figure 5:**
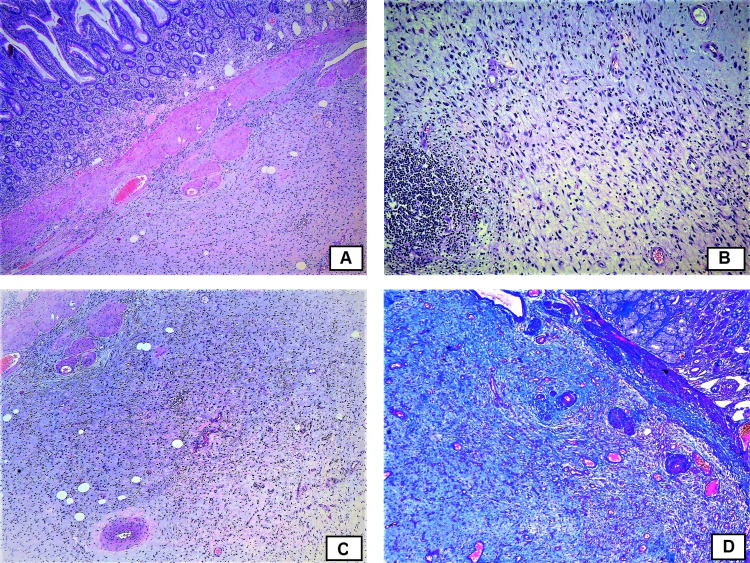
Histological examination. A - preserved mucosal layer with slightly hypertrophic muscularis mucosae, original magnification x40, hematoxylin and eosin (H&E) stain; B – submucosal layer with granulation tissue, original magnification x100, H&E stain; C – submucosal layer, original magnification x40, H&E stain; D – mucosa and submucosa, original magnificiation x40; Azan stain.

**Figure 6:**
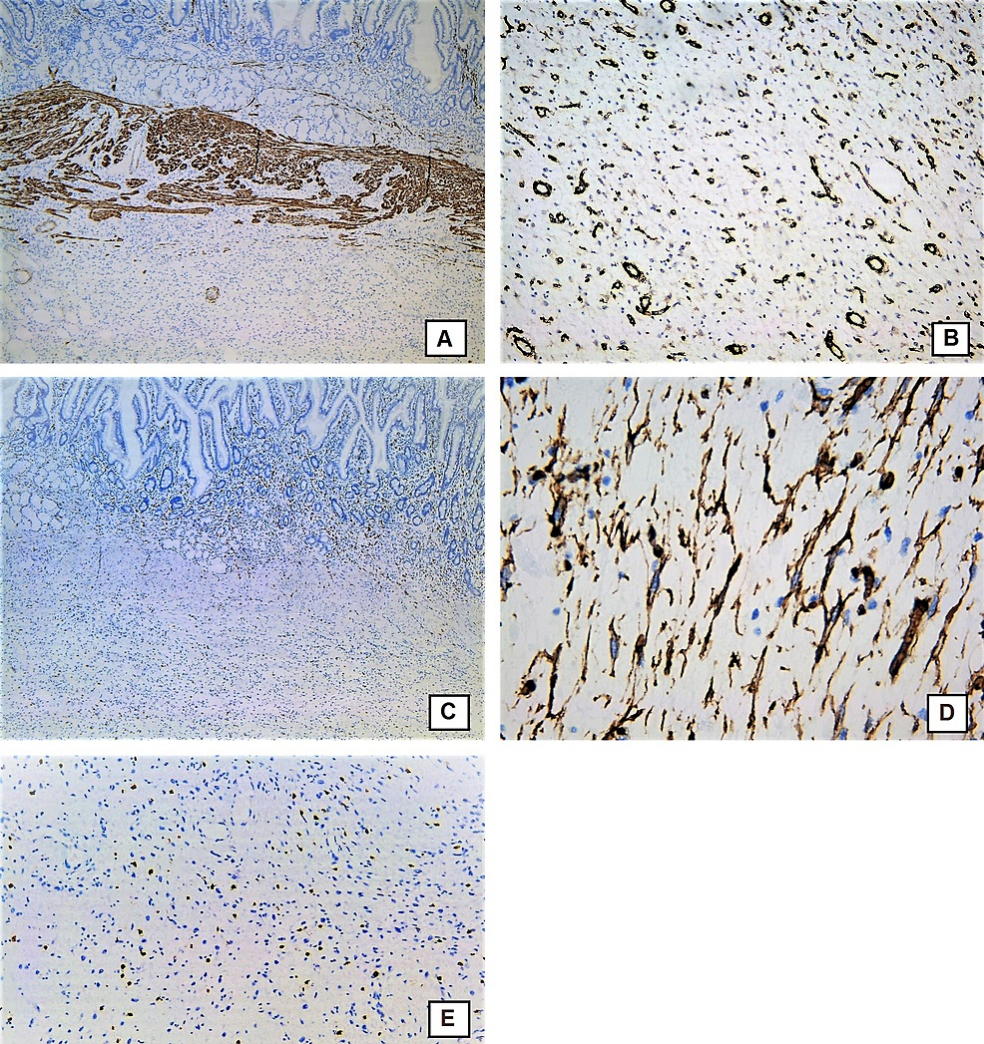
Immunohistochemistry. A – immunohistochemical stain with anti-CD117 antibody, positive expression in mast cells and negative expression in the elongated spindle cells original magnification x40; B - immunohistochemical stain with anti-CD117 antibody, original magnification x100; C - immunohistochemical stain with anti-smooth muscle actin (SMA), original magnification x100, positive expression the wall of the blood vessels and in the spindle cell, placed in the submucosa; D - immunohistochemical stain with anti Desmin, original magnification x40 – positive expression in the muscularis mucosa, negative in the submucosal lesion; E – Immunohistochemical stain with anti CD34, positive expression in the blood vessels and in the spindle shaped cells in the submucosa, original magnification x200.

Histological examination showed a wall of the duodenum with preserved mucosal layer; in the lamina propria, there was a presence of moderate infiltration of lymphocytes, plasma cells, and single eosinophils with slightly hypertrophic muscularis mucosa (Figure 5). In the submucosa, there was a proliferation of small caliber thin-walled blood vessels, elongated spindle-shaped cells with a moderately pronounced infiltration of eosinophils, plasma cells, and lymphocytes with the formation of several lymphoid follicles, edematous granulation-like stroma with lipomatosis, and hypotrophic, reduced muscularis propria. There was no cellular atypia. Immunohistochemistry was performed. Immunostaining for CD117 showed positive expression in mast cells and negative expression in the elongated spindle cells which excluded gastrointestinal stromal tumor (GIST) (Figures 6A-6B). Immunostaining for smooth muscle actin (SMA) revealed positive expression in the wall of blood vessels and in some of the spindle cells (Figure 6C). There was no positive expression with antibody against desmin (Figure 6D). Immunostaining for CD34 showed positive expression in the blood vessels and in the spindle cells (Figure 6E).

## Discussion

Vanek was the first author who described this lesion in the stomach as “gastric submucosal granuloma with eosinophilic infiltration“ in 1949 [1]. Since then, many other names have been used in the literature. The most favorable term that is used nowadays is “inflammatory fibroid polyp”. It was introduced first by Helwig and Ranier in 1952 [2].

The duodenal localization for neoplasia is rare. Based on the study by Bal et al. [3], most commonly primary duodenal neoplasia consists of epithelial tumors such as adenomas, followed by lymphoproliferative tumors, mesenchymal tumors, and neuroendocrine tumors (Table 1) [4].

**Table 1:** Histological classification of duodenal polyps.

Epithelial	Submucosal	Heterotopic	Hamartomas	External	Others
Adenomas	Leiomyoma	Gastric metaplasia or heterotomia	Brunner’s glands hamartoma/hyperplasia	Local - Pancreatic carcinoma, biliary tumors	Amyloid
	Lipoma		Solitary Peutz-Jegher polyp	Distant – metastasis from lung/breast carcinoma	Tuberculosis
	Inflammatory fibroid polyps		Peutz-Jeghers syndrome		
	Carcinoid		Juvenile polyp		
	Stromal tumors (GIST)				

IFPs are considered rare and more than 70% of them are of gastric origin. Other locations are small bowel (most common in the ileum), colon, gallbladder, oesophagus, duodenum, appendix, and rarest in the rectum. In the duodenum, IFPs occur less than 1% of cases [5].

Biological behavior and rate of growth of IFP are not described in the literature probably due to immediate treatment after they are diagnosed. In our case, due to the COVID-19 pandemic and cancelation of elective surgery, we observed that the polyp has doubled in size in eight months.

Clinical presentation may vary from asymptomatic to non-specific symptoms of upper abdominal pain, occult hemorrhage, anemia, dyspepsia [6]. Duodenal polyps are diagnosed incidentally. They are found on EGD, CT. No specific CT criteria are available to diagnose IFP of the duodenum [7]. Grossly IFP can present as solitary sessile, or in rare cases as pedunculated polyp arising from the stomach or intestinal wall and protruding into the lumen. The lesion is usually covered by mucosa which could be intact or with areas of ulceration [8]. The diameter of the tumor lesions ranges from 0.2 to 8 cm [9].

GI endoscopy reveals protruding intramural lesions with a smooth and often ulcerated mucosa. Differential diagnosis should be made with GIST and submucosal lipoma, due to similar macroscopic appearance [10]. Endoscopic biopsies are unhelpful for correct diagnosis [11]. Further diagnostic evaluation could be performed with the help of EUS to establish the localization of the tumor in the duodenal layer whether it is epithelial, submucosal, or transmural. On EUS IFP lesions appear as hypo-echogenic and homogeneous to heterogeneous formation, originating with indistinct margin from the submucosal layer. In contrast, GIST neoplasia is transmural, lipoma has well-circumscribed borders and homogeneous echoic structure [12].

Treatment of duodenal lesions could bear high procedural risks due to the thin wall and rich blood supply. Endoscopic polypectomy often is the ideal technique when the duodenal polyp is pedunculated. Given that IFP originates from the submucosal layer, it is often sessile and endoscopic resections hide greater risks of duodenal perforations, incomplete resection, and following local recurrence [13]. Considering all these factors, surgery provides the lowest risk for procedure-related complications.

On histological examination, IFP is composed of fibrous connective tissue stroma with a proliferation of variable-sized blood vessels, and with diffuse inflammatory infiltrate - eosinophils, plasma cells, lymphocytes, macrophages, and mast cells. Cellular atypia and high mitotic activity are not reported in the literature [9].

Diseases like GIST, inflammatory myofibroblastic tumor, and inflammatory polyp of Crohn’s disease must be considered in the differential diagnosis of IFP because they could be observed in the same location. GIST is a malignant tumor composed of plump spindle cells, with pale, eosinophilic cytoplasm. The spindle cells are CD117 positive [14]. In our case, there was a positive expression for CD117 only in mast cells and negative expression in the spindle cells, hence GIST diagnosis was rejected. Inflammatory myofibroblastic tumor is usually observed in children and has similar histology - plasma cells, lymphocytes, and eosinophils are observed with less prominent blood vessels admixed with a proliferation of spindle-shaped fibroblasts/myofibroblasts. Immunohistochemically the cells in inflammatory myofibroblastic tumors are positive for SMA which usually confirms its myofibroblastic nature, positive for Desmin and ALK and there is a negative expression for CD34 [15]. In our case, there was a negative expression for Desmin and positive for CD34. Another specific feature of inflammatory myofibroblastic tumors is the fact that this tumor can recur [15]. Inflammatory polyps of Crohn’s disease in the small intestine may have similar histology but usually, there is different clinical history and findings.

## Conclusions

IFP seen in the duodenum is a rare disease location with non-specific clinical presentation. Definitive preoperative diagnosis is commonly not possible and it is made after surgical resection of a suspected tumor lesion. Biological behavior is unknown while in our case, the polyp has doubled in size in eight months. Diseases like GIST, inflammatory myofibroblastic tumor, and inflammatory polyp of Crohn’s disease must be considered in the differential diagnosis of IFP because they could be observed in the same location. The final diagnosis cannot be done without complete surgical excision and the use of immunohistochemistry is essential
